# Ontogenetic loops in habitat use highlight the importance of littoral habitats for early life-stages of oceanic fishes in temperate waters

**DOI:** 10.1038/srep42709

**Published:** 2017-02-16

**Authors:** Patrick Polte, Paul Kotterba, Dorothee Moll, Lena von Nordheim

**Affiliations:** 1 Thünen-Institute of Baltic Sea Fisheries, Alter Hafen Süd 2, 18069 Rostock, Germany; 2 Institute of Hydrobiology and Fisheries Science, University of Hamburg, Olbersweg 24, 22767 Hamburg, Germany

## Abstract

General concepts of larval fish ecology in temperate oceans predominantly associate dispersal and survival to exogenous mechanisms such as passive drift along ocean currents. However, for tropical reef fish larvae and species in inland freshwater systems behavioural aspects of habitat selection are evidently important components of dispersal. This study is focused on larval Atlantic herring (*Clupea harengus*) distribution in a Baltic Sea retention area, free of lunar tides and directed current regimes, considered as a natural mesocosm. A Lorenz curve originally applied in socio-economics to describe demographic income distribution was adapted to a 20 year time-series of weekly larval herring distribution, revealing size-dependent spatial homogeneity. Additional quantitative sampling of distinct larval development stages across pelagic and littoral areas uncovered a loop in habitat use during larval ontogeny, revealing a key role of shallow littoral waters. With increasing rates of coastal change, our findings emphasize the importance of the littoral zone when considering reproduction of pelagic, ocean-going fish species; highlighting a need for more sensitive management of regional coastal zones.

Understanding dispersal mechanisms of larval fish is vitally important in determining whether fish during early life stages can grow and survive in marine habitats, a prerequisite to the successful recruitment of a population. For temperate waters, some general concepts or paradigms postulated decades ago set the direction of modern fishery science. Dispersal and survival of larval fish has been widely attributed to: passive drift along prevailing wind and current regimes (aberrant drift hypothesis^1^; stable retention hypothesis^2^), accumulation of larvae due to thermoclines and haloclines in stratified water bodies (stable ocean hypothesis^3^), resulting in spatial and temporal overlap with suitable planktonic prey (match-mismatch hypothesis^4^,^5^), particularly at the transition between yolk consumption and exogenous feeding (critical period hypothesis^6^). Most of these principal concepts were developed based on the early life history ecology of small pelagic fishes, such as the clupeid species with a rather cryptic, translucent larval morphology. Studies have rarely discriminated between larval development stages although mobility and physico-chemical tolerance ranges can differ significantly in the early stages of life^7^,^8^. Although behavioural traits and active habitat selection are important drivers of larval settlement for many tropical reef fish^9^,^10^ and are increasingly implemented in coastal zone management^11^, these behavioural aspects are not often considered as mechanisms of larval dispersal in temperate waters^12^. However, in riverine systems including those with significant current velocities, larval dispersal appears to be not entirely passive but has an active, behavioural component^13^. According to the distinct morphology of consecutive development stages it can be assumed that active habitat selection becomes increasingly pronounced along the early ontogeny and laboratory experiments have shown a significant increase of mobility in successive stages of larval Atlantic herring (*Clupea harengus*)^7^.

If active habitat selection is involved in larval dispersal of major fishery species such as herring, this would have significant impact on the interpretation and application of each of the paradigms outlined above. The overlap of larval distribution with suitable planktonic prey fields might not be exclusively determined by large scale oceanography but would also be influenced by the directed movement of larvae. A potential shift from passive to active dispersal at an advanced larval stage prior to metamorphosis to the juvenile fish would render dispersal models, integrated over the entire larval phase, imprecise to an unknown extent. However, the incorporation of behavioural aspects would potentially improve Parameterisation of such models.

In the Northeast Atlantic Ocean many commercially important fish species, such as plaice (*Pleuronectes platessa*) and Atlantic cod (*Gadus morhua*), recruit from pelagic eggs and larvae, moving towards coastal nursery grounds as post-larval juveniles^14^,^15^,^16^. For herring that spawn benthic eggs in coastal areas, a reverse dispersal of hatched larvae from inshore and coastal shelf bank spawning grounds towards offshore habitats has been documented (e.g. refs 17 and 18). This unidirectional habitat shift might be contradicted by active habitat selection when i.e. larval fish frequent littoral zones for retention as is known from riverine fish larvae^19^. Although current velocities and hydrodynamic forcing in rivers can be at magnitudes higher than in the ocean, limnetic fish larvae do not necessarily display linear unidirectional habitat use following the direction of the flow but primarily forage in sheltered retention zones in the littoral zone of river beds^13^,^20^. In freshwater systems, inshore retention areas and specifically the littoral zone with sheltered hydrodynamic conditions and macrophyte cover allows for better foraging^13^,^20^, increased prey density (e.g. refs 21 and 22) and decreased exposure to predators^23^,^24^. The function of habitats located in the upper littoral zone for larvae of ocean-going species such as herring was reported on by Urho and Hildén^25^ 25 years ago.

They attributed a decrease in abundance of larval herring in the outer coastal waters of the Baltic Sea to inshore migration rather than to mortality and suggested that knowledge of the spatial distribution of herring larvae must be broadened to understand the drivers of year-class strength.

The present study investigated habitat use of larval Atlantic herring in the Baltic Sea where the relative calm hydrology of retention areas provides a suitable model system to study stage-dependent habitat selection of differing larval development stages independent from large-scale physico-chemical stratification or the impact of major current regimes.

We adapted demographic distribution analysis from socio-economics to a 20 year time series of weekly larval distribution supplemented by field studies of stage-specific larval abundance in littoral and pelagic habitats. The objectives of the study were to test the hypotheses that i) spatial homogeneity of distribution is stage-specific, ii) vertical stratification of herring larvae occurring in shallow, well mixed water bodies point to an active positioning of larvae in the water column and iii) stage-specific shifts in habitat use are not unidirectional on the inshore-offshore gradient but the littoral zone is an important retention area for advanced larval stages.

## Results

### Size-specific larvae distribution

**Figure 1:**
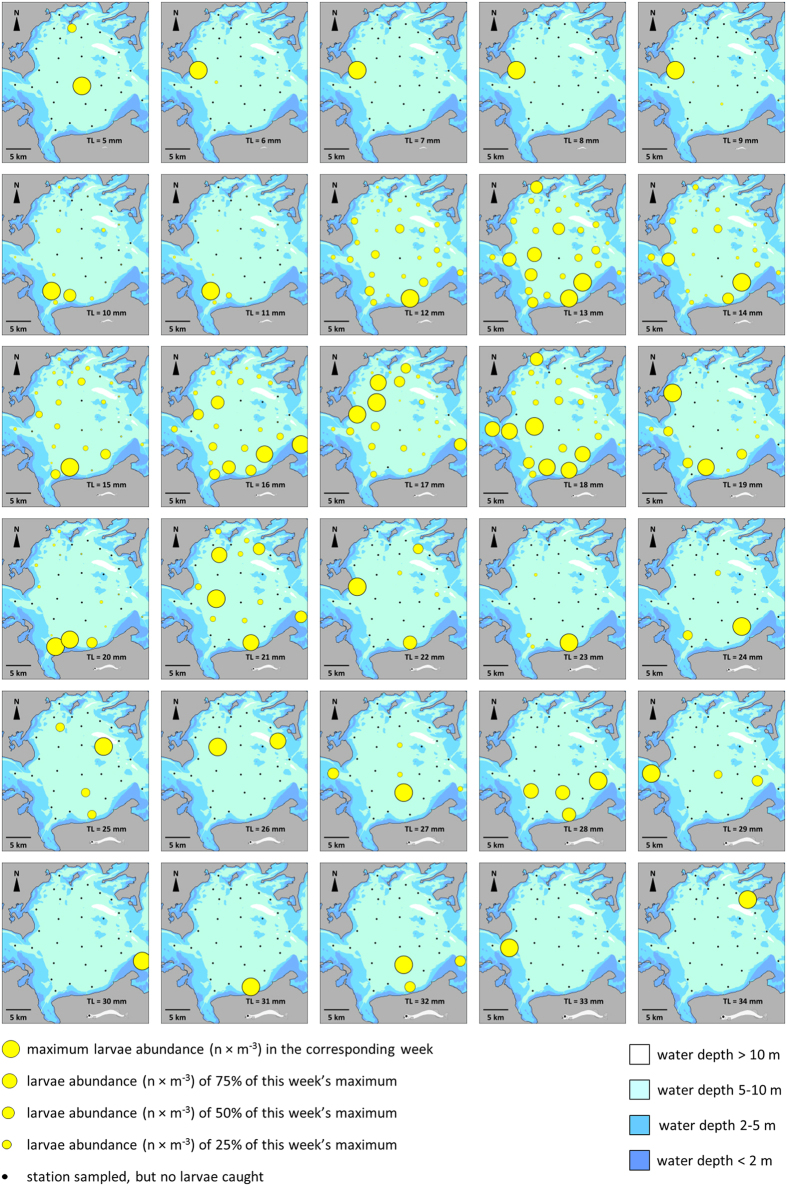
Relative distribution of larval herring size classes (mm total length) during weeks of their maximum absolute abundance in 2011. Data are based on the number of larvae per m^3^ filtered water. Size of yellow circles reflects the relative proportion of maximum abundance observed in the particular week. Source of elevation data: Federal Maritime and Hydrographic Agency (BSH), Germany. Maps were created using Esri^®^ ArcGIS 10.2 software package (URL: www.esri.com).

**Figure 2:**
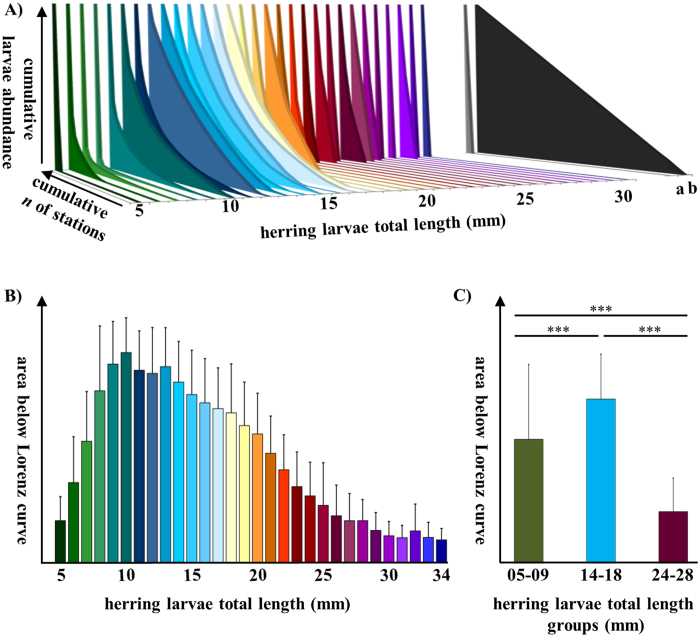
Size-specific spatial distribution homogeneity of larval herring. (**A**) Spatial homogeneity of herring larvae of different size classes (5–34 mm) plotted in a Lorenz curve design (exemplarily shown for 2011): “a” and “b” represent theoretical curves for maximum heterogeneity and homogeneity, respectively. (**B**) Mean Homogeneity of larval distribution for each 1 mm length class aggregated for the years 1992–2014. Bars represent arithmetic means, error bars represent standard deviations. (**C**) Mean area (and standard deviations) below Lorenz curve for selected larvae length groups. Horizontal bars with asterisks indicate significant differences (***represents a significance level of *p* ≤ 0.001).

We found strong size-dependent differences in the spatial distribution of herring larvae in the study area (Fig. 1). Weekly distribution data of each larval length group (mm TL) were used to visualize the level of distribution homogeneity by a Lorenz curve (Fig. 2A). The size classes assigned to certain larval development stages based on information from the literature^7^,^26^ were found clearly reflected by the post-hoc test (Games-Howell test) of Lorenz curve areas of each mm size class (see Table S5 for details). Therefore, shifts in distribution homogeneity could be assigned to particular size classes which translate to developmental stage with sufficient certainty. Figure 2B reflects this pattern by showing aggregated measures of homogeneity for the years 1992 to 2014 represented by the mean areas below the corresponding Lorenz curves. Kruskal-Wallis test confirmed highly significant differences in spatial distribution homogeneity of distinct length classes (χ^2^ = 477.447, df = 29, *p* < 0.001). While there was a gradual increase of distribution homogeneity observed for the larvae prior to the first feeding stage (5–9 mm), larger larvae can be assigned to sharply delimited groups along their homogeneity of spatial distribution (Fig. 2B, Table S5). Life-stage associated groups of length classes were also significantly different (Fig. 2C; χ^2^ = 140.704, df = 2, *p* < 0.001).

Larval size classes from 5–9 mm (yolk-sac) all occurred on a single patch resulting in a very heterogeneous spatial distribution over the entire sampling area. The intermediate stages represented by the size class of 14–18 mm (flexion stage) were distributed homogenously throughout the bay. The advanced stages of larvae in size class 24–28 mm (post-flexion) stage showed a high spatial heterogeneity.

### Larval habitat utilization

**Figure 3:**
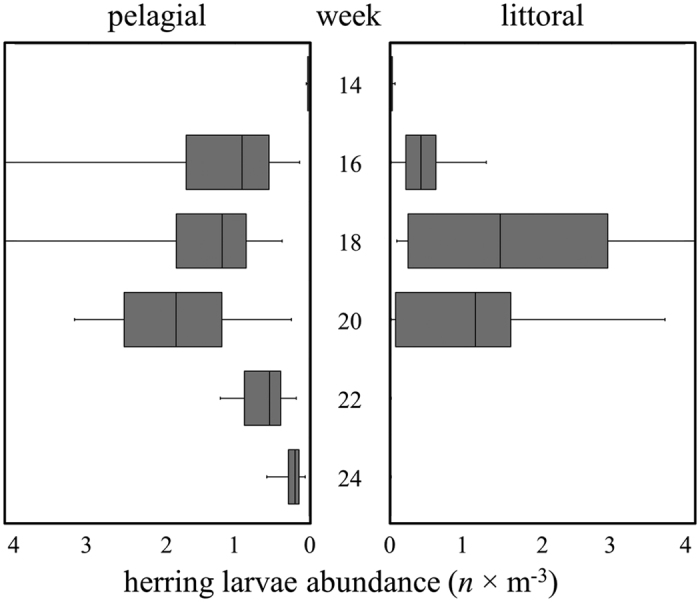
Herring larvae abundances in the pelagic zone of Greifswald Bay (left panel) during selected weeks in 2011 compared with larvae abundances in the littoral zone (right panel) in corresponding weeks. Data are presented in boxplots with boxes reaching from the 0.25 quantile to 0.75 quantile (containing the median – given as horizontal line). Whiskers represent the absolute maximum and minimum values, respectively.

Quantitative sampling of larval herring in the pelagic zone and the respective littoral zones of the bay resulted in comparable numbers in both habitats (Fig. 3). However, the size distribution differed slightly (Fig. SI 6). The majority of larvae found in the littoral zone during the sampling season (calendar week 14–24, 2011) was composed of yolk sac larvae (mean length 8.1 mm, SD = 2.3 mm). Throughout the season the abundance in the littoral zone (Fig. 3) peaked about two weeks earlier (calendar week 18) than in the pelagic zone (calendar week 20). With the passing of the season growing numbers of pre-flexion larvae (size 10–14 mm) could be observed in the littoral habitat but with increasing size their distribution shifted towards the pelagic zone. The mean size of larvae found in the pelagic zone during the sampling duration was 9.9 mm (SD = 3.4 mm).

### Vertical distribution

**Figure 4:**
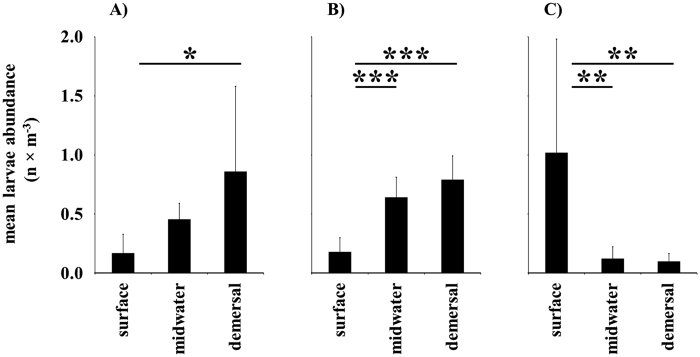
Depth distribution of herring larvae (14–18 mm TL) on different sampling stations in the study area. Bars represent mean values while the standard deviation is given by error bars (*n* = 6 hauls for each group). ‘Surface’ indicates larvae abundance close to the sea surface, ‘demersal’ refers to the sampling depth of 1 m above the sea bottom and midwater to the sampling in between. (**A**) Northwestern station located in the narrow sound between the Island of Rügen and the mainland (maximum sampling depth = 9 m). (**B**) Station located at the western edge of Greifswald Bay (maximum sampling depth = 4 m). (**C**) Station at the southern coast of the bay (maximum sampling depth = 7 m). Horizontal bars with asterisks indicate significant differences based on a one-way ANOVA and corresponding post-hoc tests (Tukey HSD-test; significance levels are given as: *p ≤ 0.05; **p ≤ 0.01, ***p ≤ 0.001).

Pre-flexion and flexion stages (14–18 mm) of herring larvae were relatively homogeneously distributed within the bay but exhibited a significantly differentiated vertical distribution even in very shallow areas of the bay (Fig. 4). However, the orientation of vertical distribution patterns was not consistent for all stations: While larvae seemed to aggregate in bottom waters at two stations (Fig. 4A,B), the pattern was reversed in the third station (Fig. 4C). The cloudiness differed significantly (ANOVA: F_(1,16)_ = 6.938; *p* = 0.018) between the sampling times of the first two stations (^2^/_8_ ± ^3^/_8_) compared to the conditions at the third station (^5^/_8_ ± ^2^/_8_). CTD profiles recorded in Greifswald Bay during the period of vertically explicit larvae sampling revealed no physico-chemical stratification of the water column and mean differences of surface and bottom temperatures, salinity and dissolved oxygen were marginal (Table S7).

### Distribution of post-flexion larvae

**Figure 5:**
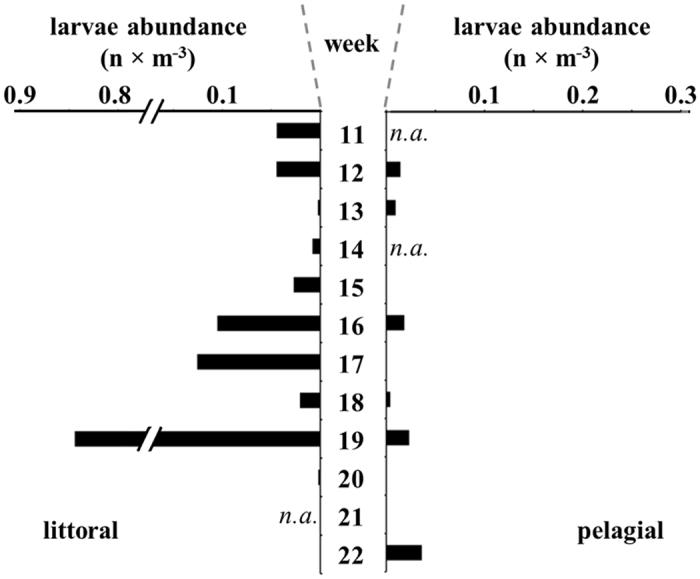
Depth dependent abundance of advanced herring larvae (>20 mm) in Greifswald Bay. Left panel shows results of weekly beach seine samples in 2015 (littoral zone), while the right panel shows the abundances derived from ring trawl catches in corresponding weeks (pelagic zone). N.a. = not analysed and sample not taken, respectively. Note the fracture of the y-axis for the littoral data.

Advanced herring larvae of >20 mm were found to form large shoals in the very shallow littoral (<0.5 m depth) of Greifswald Bay during spring 2011. Beach seine sampling in spring 2015 revealed high abundances of larvae (up to a maximum of 0.84 larvae m^−3^) in the littoral zone (Fig. 5). However, simultaneous ring trawl catches in the adjacent pelagic zone did not result in comparably high abundances (up to a maximum of 0.04 larvae × m^−3^; Fig. 5).

## Discussion

The Lorenz curve applied to the multi-decadal time series of weekly larval herring abundance revealed significant size-specific patterns in the spatial distribution of the herring larvae. Attributing larval size classes to development stages (e.g. refs 7 and 26), hatchlings (yolk sac stage) and advanced larvae (post-flexion) prior to metamorphosis showed high distribution heterogeneity, whereas the pre-flexion and flexion stage larvae were distributed more homogenously within the bay. In fish ecology, habitat requirements are generally assigned to ontogenetic levels (i.e. egg, larvae, juvenile, adult) rather than to development stages on a specific ontogenetic level. However, major fishery species in the Baltic Sea, such as cod, sprat (*Sprattus sprattus*) and herring all have complex life cycles including several larval stages that differ significantly in morphology and motility traits^7^,^27^. It is obvious that morphological and ecological differences can be more pronounced between successive larval development stages than between respective post-metamorphosis juvenile and adult stages.

Along the early herring ontogeny our results indicate a selective habitat use according to larval development stage including upper littoral and pelagic habitats of the system. After hatching, larvae in the yolk sac stage left the shore zone and moved towards pelagic habitats of the basin. However, they remained aggregated in the vicinity of the spawning beds. Larvae in the intermediate development stage (pre-flexion, flexion) were found to be increasingly dispersed throughout the pelagic zone of the bay. In contrast, fish in the advanced stages (post-flexion) were found abundant in the upper littoral zone while almost absent in the pelagic zone.

**Figure 6:**
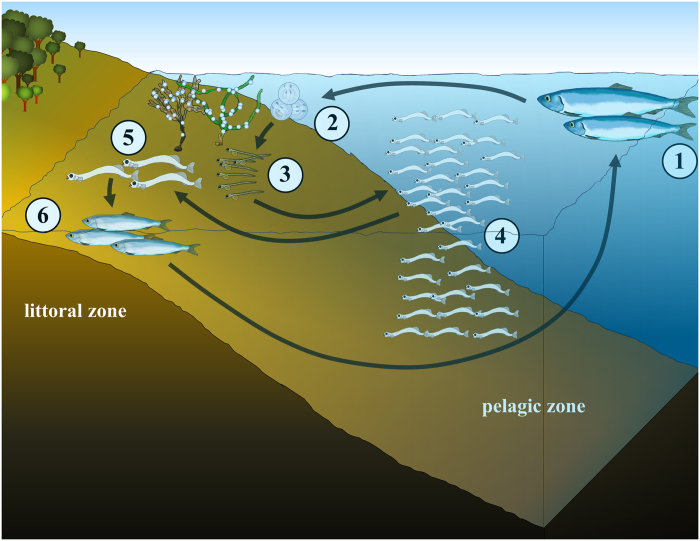
Ontogenetic habitat loop of herring in the Baltic Sea. Adult herring (1) migrate from the offshore pelagial into inshore waters to spawn their adhesive eggs on littoral substrates such as macrophytes (2). Yolk-bearing hatchlings (3) appear concentrated in the vicinity of the spawning beds, while medium sized larvae (4) are rather well horizontally distributed in the pelagic area of the bay; however their vertical distribution in the water column is significantly heterogeneous. Advanced larvae (5) return to shallow littoral areas where they remain until after their metamorphosis to the juvenile fish (6). Growing juveniles migrate to offshore areas along their development and usually recruit to the spawning group after 2–3 years.

For technical reasons, differing sampling gear had to be used in the pelagic and littoral habitats. Because of potential bias introduced by gear type no direct statistical comparison of larval abundance in both habitats could be achieved but we demonstrated relative abundances of post-flexion larvae caught by the respective gear. As the ring trawl is standard gear to sample ichthyoplankton (e.g. ref. 28), it can be suggested that the low larval numbers caught in the pelagic zone generally are a consequence of selective habitat use. Larvae at different developmental stages which have been observed repeatedly in the shore zones indicate a certain loop in habitat use (Fig. 6) that, to our best knowledge, has not yet been documented even for an otherwise intensively studied fishery species such as herring. This habitat loop is even more surprising as most studies on larval dispersal commonly describe a rather unidirectional succession of habitats during the ontogeny of young fish. Generally, larval fish movement towards near shore habitats by either passive or active dispersal mechanisms has been widely described for ocean spawning fish^29^,^30^. However, “near shore” often refers to the open water body of estuaries, bays and lagoons but rarely includes the shore zone gradient encompassing the upper littoral zone just below the low tide mark.

In the Northeast Atlantic Ocean, many fishery species recruit from pelagic eggs and larvae. Species such as plaice and Atlantic cod are thought to disperse towards coastal nursery grounds as juveniles^31^,^32^,^33^,^34^. For herring that spawn benthic eggs in the inner coastal areas, a reverse dispersal of hatched larvae towards outer coastal zones is generally assumed (e.g. refs 17 and 35). Hence the majority of larval herring surveys conducted for stock assessment purposes are performed in outer coastal waters which substantially neglects the potential larvae habitats in the shore zones. This might represent a somewhat reversed scaling issue by ignoring the contribution of small scale habitats with significant ecological function but with limited geographic extension to overall system productivity. However, stressors to larval survival in inshore retention areas e.g. by altered habitat conditions would explain the discrepancies observed in classic analyses of stock recruitment relationships (e.g. refs 36 and 37) and would help to clarify the stock-recruitment dilemma.

The example of the Western Baltic herring stock illustrates how local stressors on early life stage survival in spawning grounds and larval retention areas can affect entire population dynamics^38^. In a system without significant tidal forcing or large scale current regimes, such as the Baltic Sea, behavioural traits might be important mechanisms of larval dispersal. As littoral habitats in temperate waters are underrepresented as important habitats for larval fish by coastal zone management, there remains a potential risk that important fish resources are being affected by habitat degradation before fishes grow into size classes relevant for current stock assessment models. Our case study on the Western Baltic herring could be considered a prime example of this problem because fishery mortality of the stock was drastically decreased during the past decade due to strict quota driven fishery restrictions. However recruitment also continuously decreased during that period, driven by mortality during the early life stages^39^.

Even in well-mixed waters of the lagoon without pronounced thermoclines or haloclines, fish in the pre-flexion and flexion larval stages were not homogeneously distributed within the vertical water body. Despite site- and day-specific vertical zonation, the overall findings that larvae have shown a distinct distribution hints at the potential of these fish in the larval stages to be capable of active vertical positioning. According to Schnack^40^ the site- and date- specific differences in larval zonation patterns might be due to differing degrees of cloud cover where larvae are found closer to the surface on an overcast day. At station “C” at an intermediate depth (7 m), larvae were accumulated at the surface whereas at the other two stations they were found in mid-water or close to the sea bed. However station “C” was sampled about one week later when cloud cover was significantly higher. This distribution difference would reflect the documented observation that vertical migration is driven by light levels (e.g. refs 41 and 42). Vertical migration is an important means of dispersal as ichthyoplankton might use certain vertical current regimes for dispersal^43^. Hence, behavioural traits should be considered in parameterizing spatial dispersal models for larval herring.

The distinct larval distribution hints at an active habitat selection according to life stage. This suggests that transitional waters might offer important retention areas in the developmental stages of early herring until metamorphosis to the juvenile stage.

Since Johan Hjort^6^ postulated his critical period hypothesis it has been commonly accepted in fishery science that recruitment of fish stocks is often determined early in the larval stage of a species. The mechanisms causing most larval fish mortality are assumed to be predominantly linked with predation^44^ and food availability for early larvae^6^,^4^. Habitat-specific mortality could potentially result in inhomogeneous distribution in the early life stages. Major mortality e.g. by starving after yolk consumption, occurs over a period from 6 to 8 days^45^. Due to the high sampling frequency it is unlikely that we missed major mortality events related to habitat type. Concerning top-down control, earlier studies have shown that predation on herring larvae is of minor importance in the system^46^. Interpreting the different stage-specific patterns of distribution as being a consequence of active habitat selection, this study indicates that habitat requirements might vary according to the stage of larval development. This could introduce an undocumented suite of survival bottlenecks related to availability and connectivity of differing juvenile habitats. In addition to fishery impacts on the adult populations, coastal modification and habitat degradation might introduce significant stressors to important fish resources. An appropriate quantity of spawning stock biomass (SSB) is clearly necessary to sustain a population; however this alone does not guarantee solid recruitment. It must also be considered that variability or a decrease in recruitment is widely structured by environmental conditions including biotic factors such as predation and competition as well as variation in the physico-chemical environment (e.g. refs 47 and 48). Many of the general theories and paradigms in larval fish ecology were developed by studying the clupeid species. The commonly accepted general concepts such as the stable ocean hypothesis^3^ or the stable retention hypothesis^2^ are broadly based on the impact of the physical environment on larval dispersal. Leis^9^ questioned whether the role of behavioural patterns are important for larval dispersal and concluded that behavioural aspects are more pronounced in tropical species compared to species inhabiting colder waters, due to extrinsic physical and intrinsic physiological reasons. However, in the shallow retention areas of the Baltic Sea, our study indicates that even the fragile and cryptic clupeid larvae are able to actively select their habitats.

The ecological value of shallow temperate systems in general (e.g. ref. 49) and of littoral habitats in particular for larval fish have been only rarely incorporated in both scientific and marine policy plans, although 25 years ago Urho and Hildén^25^ described the importance of these considerations for Baltic Sea herring stocks. This gap is increasingly problematic as particularly shallow coastal habitats are exposed to the growing threat of drastically changing coastlines caused by human activity as well as numerous major environmental factors such as sea surface warming. Although the habitat function of shallow upper littoral zones as nurseries for fish communities has been broadly acknowledged in marine and freshwater systems (e.g. refs 50 and 51), their role in the larval period of “wasp waist”^52^ species in coastal food webs in temperate waters is not well understood. The findings of this study should not only provide a strong further impetus for investigation of the ecological functions and value of shore zones for early life stages of oceanic fish species, but will also encourage the implementation of these findings into current management of coastal zones.

## Methods

### Stage-specific spatial distribution homogeneity

The distribution of herring larvae was studied in the Greifswald Bay (N54°14′ E013°34′), a major spawning ground and retention area for Atlantic herring in the Western Baltic Sea (see Supporting information and Fig. S1 for details). The ‘Rügen Herring Larvae Survey’ (RHLS) is an ongoing complex stock assessment process where herring larvae are quantitatively sampled weekly at 36 stations in the bay and adjacent waters between March and July^53^ (see SI and Fig. S2A for details). Thirty stations from the RHLS were included in the present study. Stations located in the adjacent sound with a different hydrology and were excluded from the analysis. For the investigation of the spatial distribution of different herring size classes (Fig. S1), data from the years 1992 to 2014 were used to initially visualize the relative distribution during the calendar weeks of the highest abundances of each length group and each year using the Esri^®^ Geographic Information System ArcMap™ version 10.2 (see SI for further information on the selection process).

The homogeneity of spatial dispersal of the larvae was then further characterized in detail by plotting the data into a Lorenz curve^54^. We then calculated the area below the curve as a measure of data homogeneity:


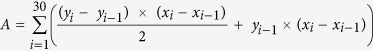


where *A* is the area below the Lorenz curve in relative dimensions, *y* represents the cumulative proportion of larvae abundance (0 ≤ *y* ≤ 1) and *x* is the cumulative proportion of sampling stations (*x* = ^1^/_30_; ^2^/_30_; ^3^/_30_;…;1). We used a Kruskal-Wallis test and corresponding post-hoc tests (Games-Howell test) to examine the differences between the larvae sizes.

### Assigning larval size classes to specific development stages

Larval herring size classes (mm TL) were assigned to particular life stage categories according to literature on western Baltic herring^7^ and central Baltic herring^26^. The transition between stages ranges between multiple millimeters in growth and differences in critical morphological traits (e.g. development of fin rays) could potentially affect larval distribution patterns. Therefore it was necessary to select size categories with definite stage classification and discard intermediate length classes to investigate stage specific habitat selection. Accordingly, larvae between 5 and 9 mm were categorised as yolk-sac-bearing hatchlings not yet feeding actively, while larvae between 14 and 18 mm were classified as having already passed the critical period of first feeding (shift from endogenous to exogenous nutrition). Larvae between 24 and 28 mm were classified as having already passed the main structuring bottlenecks of larval survival since their abundance relates linearly to the resulting numbers of juvenile fish caught in consecutive years^53^. We used a Kruskal-Wallis test and corresponding post-hoc tests (Games-Howell test) to compare the means of the distinct groups.

### Larval herring sampling in the littoral zone

Every second week between April and June in 2011, herring larvae abundances in the shallow littoral zone of the Greifswald Bay were investigated using a plankton net mounted on an epibenthos sledge (see details in SI and Fig. S2B). The data were normalized against the volume of filtered water and then compared to simultaneous RHLS catches of larvae from the pelagial of the bay.

### Vertical distribution in the pelagic zone

In April 2012, the vertical distribution of herring larvae was investigated with standardised plankton samples at three distinct depths strata on three different stations within the Greifswald Bay (Fig. S1). Sampling was repeated six times at each depth stratum at every station and larvae abundance was calculated by the volume of water filtered by the plankton net (see details in SI and Fig. S2C). Larvae abundance from each depth strata from the stations were compared by ANOVA and corresponding post-hoc tests (Tukey’s HSD test). As the level of cloudiness might affect depth stratification of the larval fish, weather data, derived from the federal meteorological service of Germany (DWD) were used to analyse the cloudiness during the depth-stratified sampling. For each sampling time, the mean hourly cloudiness (n = 9) was compared and an ANOVA was performed to test for significant differences in cloud cover. Furthermore, water probe profiles measuring conductivity, temperature and oxygen content of the water (CTD) were recorded to analyse the level of mixing in the water column. A total of 40 CTD profiles were recorded in the bay during the sampling period. We compared surface and bottom values of salinity, temperature and oxygen in order to identify possible stratification events of water masses.

### Sampling of advanced larval stages

Between March and June 2015 an additional sampling was performed weekly, specifically targeting herring larvae in advanced growth stages prior to metamorphosis. Sampling of larvae in the pelagial of the bay was conducted with a ring trawl equipped with a 1.5 mm mesh plankton net (see SI and Fig. S2D for detailed information). Sampling in the shallow littoral zone was conducted with a beach seine (mouth opening 7 m, mesh size 5 mm (Fig. S2E), towed over a distance of 100 m. The exact area fished by beach seine hauls was determined by Global Positioning System.

Principle environmental parameters such as sea temperature, salinity, oxygen saturation and weather data were recorded at the same time as the field sampling.

All field samplings of larval fish were conducted under current licenses for wild fish sampling according to Mecklenburg-West Pomeranian (Germany) fishery law (§ 11 LFischG, Landesamt für Landwirtschaft, Lebensmittelsicherheit und Fischerei, Mecklenburg-Vorpommern).

## Additional Information

**How to cite this article**: Polte, P. *et al*. Ontogenetic loops in habitat use highlight the importance of littoral habitats for early life-stages of oceanic fishes in temperate waters. *Sci. Rep.* **7**, 42709; doi: 10.1038/srep42709 (2017).

**Publisher's note:** Springer Nature remains neutral with regard to jurisdictional claims in published maps and institutional affiliations.

## Supplementary Material

Supplementary Information
