# Supplementary material for: Ontogenetic loops in habitat use highlight the importance of littoral habitats for early life-stages of oceanic fishes in temperate waters
Source: Sci Rep. 2017 Feb 16;7:42709. doi: 10.1038/srep42709 (PMC5311983; doi:10.1038/srep42709)
Supplement: Supplementary Information [file srep42709-s1.doc]

# Supporting Information

# Ontogenetic loops in habitat use highlight the importance of littoral habitats for early life-stages of oceanic fishes in temperate waters

Patrick Polte^1*^, Paul Kotterba^1^, Dorothee Moll^1,2^, Lena von Nordheim^1,2^

1. *Thuenen Institute of Baltic Sea Fisheries, Alter Hafen Sued 2, 18069 Rostock, Germany*
2. *Institute of Hydrobiology and Fisheries Science, University of Hamburg, Olbersweg 24, 22767 Hamburg, Germany*

**Corresponding Author:* [*patrick.polte@thuenen.de*](mailto:patrick.polte@thuenen.de)*, phone: +49 (0) 381 8116-103, fax: +49 (0) 381 8116-199*

*Study area*

Greifswald Bay located at the southern coast of the Baltic Sea is a shallow (mean depth of 5.6 m), mesohaline lagoon unaffected by any significant lunar tides (Fig. S1). Sea level amplitudes are irregular and mainly wind driven and the water exchange to the adjacent Baltic Sea is limited to a broad but shallow entrance in the east and a deeper but narrow sound in Northwestern part of the bay. The lagoon includes an area of approximately 514 km² and is considered to be a major spawning ground of spring-spawning herring in the Western Baltic Sea. Between March and May, herring immigrate into the bay in massive cohorts for spawning attaching adhesive eggs to benthic substrates such as submerged aquatic vegetation.

*Rügen Herring Larvae Survey (RHLS)*

Since the bay is assumed to provide an important retention area for herring larvae, a regular ichthyoplankton survey was initiated in the 1970s to examine the inter-annual dynamics of herring larvae production. Since 1992, the survey consisted of a grid of 36 stations that were sampled weekly using a Bongo net with a mesh size of 335 µm and a round net opening of 60 cm in diameter. Catches with the Bongo net were conducted with oblique hauls at a speed of approximately 2 knots to assure a representative sampling of the entire water column from the surface down to 1 meter above the sea bottom (Fig. S2A). Only one of the two Plankton nets in the Bongo setup was used for the regular estimation of herring larvae abundance, while samples from the second one are usually used for case studies or additional analyses. The volume of filtered water was measured with mechanical and digital flowmeters (Hydrobios) installed in the center of each net. Each sampling has regularly been complemented by the recording of weather conditions, as well as turbidity, water temperatures, salinity and dissolved oxygen saturation.

Pilot studies on herring larvae abundances revealed that stations sampled in the narrow sound connecting the bay with the Baltic Sea (“Strelasund”) and in the “Peene river” mouth in the south eastern part of the bay represent hydrologically different systems compared to Greifswald Bay with significant differences in physico-chemical conditions (Polte, unpublished). Therefore, only data from the 30 stations located within the bay were included in this study (Fig. S1).

*Size-specific larvae distribution*

The spatial distribution of each mm length group (total length = TL) of herring larvae between 5 and 34 mm was investigated using length distribution data derived from the RHLS data set. Detailed information on sample processing and larvae measurements is presented in Oeberst et al.^1^. For each mm-length group (TL) in every year, we selected the calendar week of highest mean abundance for a certain size class. Figure S3 exemplarily illustrates the selection of calendar weeks used for the year 2011. To verify the approach using maximum abundance as base line for distribution homogeneity, we conducted a similar analysis using the average abundance of the entire sampling period. Both analyses resulted in the same general pattern of spatial size-specific distribution homogeneity (Fig. S4). However, using the average-based approach resulted in an increase of zero values particularly for the more heterogeneously distributed size classes. Hence the maximum-approach was applied for further analyses.

Figure S3 also indicates, that the data set on larger larvae includes two different cohorts. To exclude cohort specific effects on our results, we compared the large larvae (25-34 mm) of both cohorts regarding their distribution homogeneity and found no significant differences (Mann-Whitney test, *U*=48.0; *N*=20; *p*=0.88). Hence we pooled the data of both cohorts to improve the sample size for these length groups.

*Littoral sampling of herring larvae*

In 2011, a modified epibenthos sledge equipped with a 500 µm Plankton net (Fig. S2B) was used to investigate the herring larvae abundance in a shallow littoral area in Greifswald Bay known to include herring spawning beds (Fig. S1). The sampling was performed by boat in every second week between April and June. Towing distance and filtered volume estimations were performed using a mechanical flowmeter (Hydrobios) installed in the center of the net and a handheld Global Positioning System unit (Garmin Vista HcX). The sample replicates varied in numbers between the weeks because of differing weather conditions and logistic limitations (4 ≤ n ≤ 18).

*Vertical distribution of herring larvae*

In 2012, we sampled herring larvae at three different stations in the bay and the adjacent “Strelasund” (Fig. S1) in order to investigate the vertical distribution of larvae in the water column. The sampling was performed on three different depths zones on each station: 1m below the surface, 1 meter above the sea bottom and in midwater. The Plankton net was lowered down to the depth of interest and then towed horizontally for 5 minutes before it was recovered (Fig. S2C). For each depth zone, the sampling included 6 replicates, resulting in a total of 18 samples per station (6 replicates × 3 depth zones). Larval abundance was calculated per volume and then compared to each other.

*Spatial distribution of advanced larval stages*

Between March and June 2015 (calendar weeks 11-22), an additional weekly sampling was conducted particularly targeting larger herring larvae. The pelagial of the bay was sampled with a ring trawl of 1 meter in diameter equipped with an ichthyoplankton net (mesh size 1.5 mm, Figure S2D). The net was towed at a speed of approximately 3 knots and for 1 minute at every meter step from the surface down to 1 meter above the sea bottom. With its larger opening and the increased towing speed compared to the Bongo net, the ring trawl is generally accepted to be more appropriate to catch larger fish larvae. Simultaneously, the littoral zone at the southern coast of the bay was sampled with a beach seine of 7 m opening and a mesh size of 5 mm (Fig. S2E). The beach seine was towed manually by two persons in a direction parallel to the shore line at a depth of approximately 1.0 meter. The towing distance was measured with a handheld GPS unit (Garmin Vista HcX). Two hauls in opposite directions were conducted at each sampling day in order to compensate for tow-direction related effects on the catch compositions (wind, waves and currents). Consequently, both catches were aggregated and treated as a single sample.

All statistical analyses were performed using SPSS 17.0 software. All post-hoc tests included a Bonferroni correction of significance levels to counteract cumulating type I error.

**References**

1 Oeberst, R. *et al.* When is year-class strength determined in western Baltic herring? *ICES Journal of Marine Science* **66**: 1667–1672. doi:10.1093/icesjms/fsp143, 6 p. (2009).

**Figure & table legend (supporting information)**

Figure S1. Location of Greifswald Bay in the Baltic Sea (BS). NS = North Sea. Standard stations of the Rügen Herring Larvae Survey (1992-2014) are shown in the lower figure of the bay and labelled corresponding to how the individual stations were included in this study:
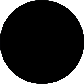
 indicate survey stations sampled weekly during the herring larvae seasons.
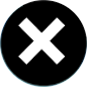
 are RHLS standard stations but not considered for the homogeneity analysis due to differing hydrology.
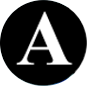
,
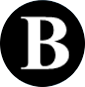
,
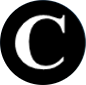
 represent RHLS standard stations selected for the 2012 case study on vertical larvae distribution (3 depth strata/station, n = 6/stratum).
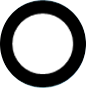
 represent stations sampled weekly during spring 2015 with a ring trawl for advanced larval size classes.
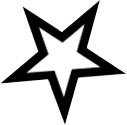
 represent the shallow littoral site where larvae were sampled with an epibenthos sledge (every second week in 2011) and a beach seine (weekly in 2015). Source of bathymetry data: Federal Maritime and Hydrographic Agency of Germany (BSH). Maps were created using Esri® ArcGIS 10.2 software package (URL: [www.esri.com](http://www.esri.com/)).

**Figure S2.** Overview of sampling procedures applied in the study. **A)** Schematic illustration of the standard sampling procedure of the RHLS sampling. At each station an oblique haul of the Bongo net is conducted down to 1 meter above the sea bottom. At each 1 meter step, the net is being towed horizontally for 30 seconds before it is lowered down for another meter. **B)** Modified epibenthos sledge equipped with a 500 µm Plankton net and a net opening of 56 × 40 cm (0.224m²). The cod end bucket is supplied with a 335µm mesh net. In the center of the net mouth, a mechanical flowmeter was installed to estimate the actual tow distance (not shown here). **C)** Schematic illustration of the sampling of herring larvae at a certain depth. This type of sampling was performed at three different depths strata with 6 replicates each resulting in 3 × 6 = 18 samples per station. **D)** Schematic illustration of the ring trawl sampling of large herring larvae in the pelagial zone of Greifswald bay. **E)** *upper panel:* Schematic illustration of the beach seine applied to catch large larvae within the littoral zone of Greifswald Bay. *lower panel:* picture taken during the closing of the net at the end of the sampling process.

**Figure S3.** Illustration exemplarily showing the selection of calendar weeks in 2011 used for the analysis of spatial distribution homogeneity. For each length group, the week of highest abundance was selected (crossed fields), providing that all stations (*n*=30) have been sampled during that particular week. This selection has been done for all years available (1992-2014). The color ramp indicates the relative abundance of each herring larvae length class during all weeks.

**Figure S4.** Size-specific spatial homogeneity of herring larvae in Greifswald Bay given as mean area (bars) below the Lorenz curve for the years 1992-2014 (error bars = standard deviation). A) Data selection (to be included in the analyses) based on the weeks of highest abundances of each length group in every year; B) Data selection based on the weeks of mean abundances.

**Figure S5**. Comparison of spatial distribution homogeneity of distinct herring larvae size classes (5-34 mm) for the years 1992-2014. Upper right part shows actual *p*-values of each post-hoc comparison (Games-Howell test). Lower left part represents a color ramp-based illustration of the pairwise comparisons between the different length groups.

**Figure S6:** Abundances of distinct size classes of herring larvae in the pelagic zone of Greifswald Bay (left panel) in selected weeks of 2011 compared with larvae abundances in the littoral (right panel) during corresponding weeks. Data are shown in boxplots with boxes reaching from the 0.25 quantile to 0.75 quantile (containing the median – given as horizontal line). Whiskers represent the absolute maximum and minimum values, respectively. Total length classes: 0509 = 5-9 mm; 1014 = 10-14 mm; 1519 = 15-19 mm; 2024 = 20-24 mm; 25++ = > 25 mm total length.

**Table S7:** CTD data of main physical variables comparing surface with bottom waters during the sampling period. Presented are mean values of water temperatures, salinity and dissolved oxygen. Mean differences between surface and bottom waters result from a pairwise analysis and the respective variability coefficients are presented.

Figure S1


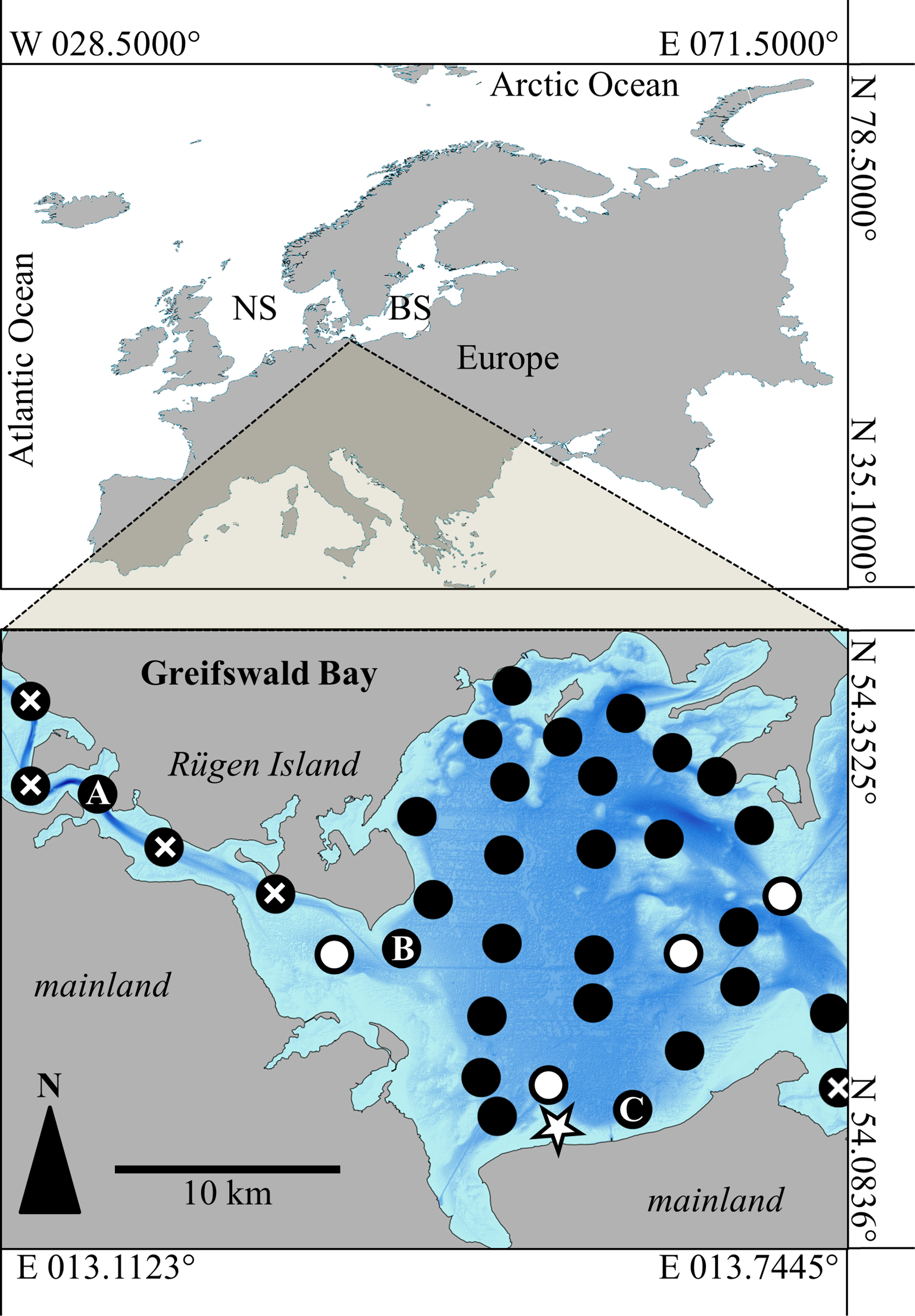


**Figure S2**


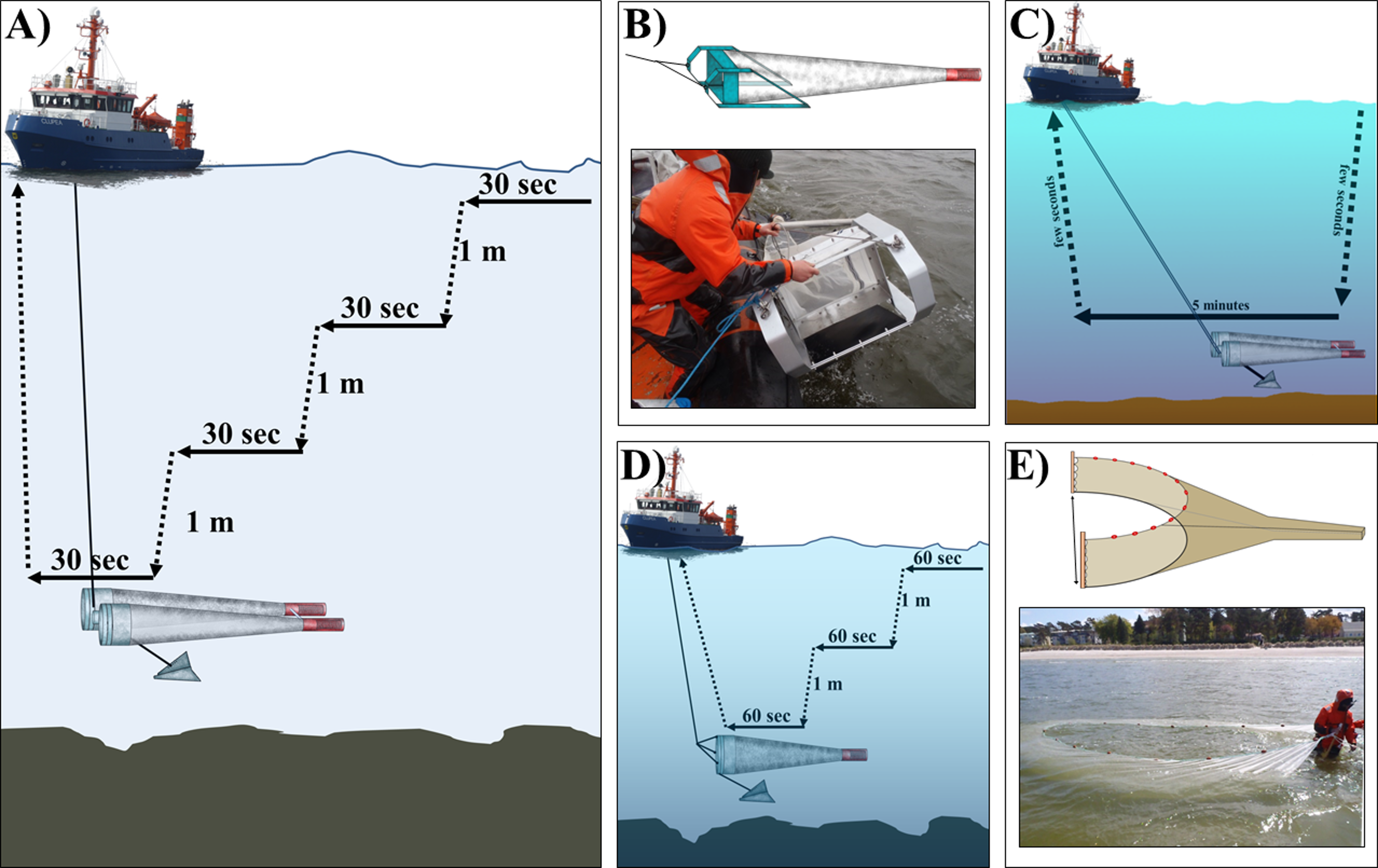


**Figure S3**


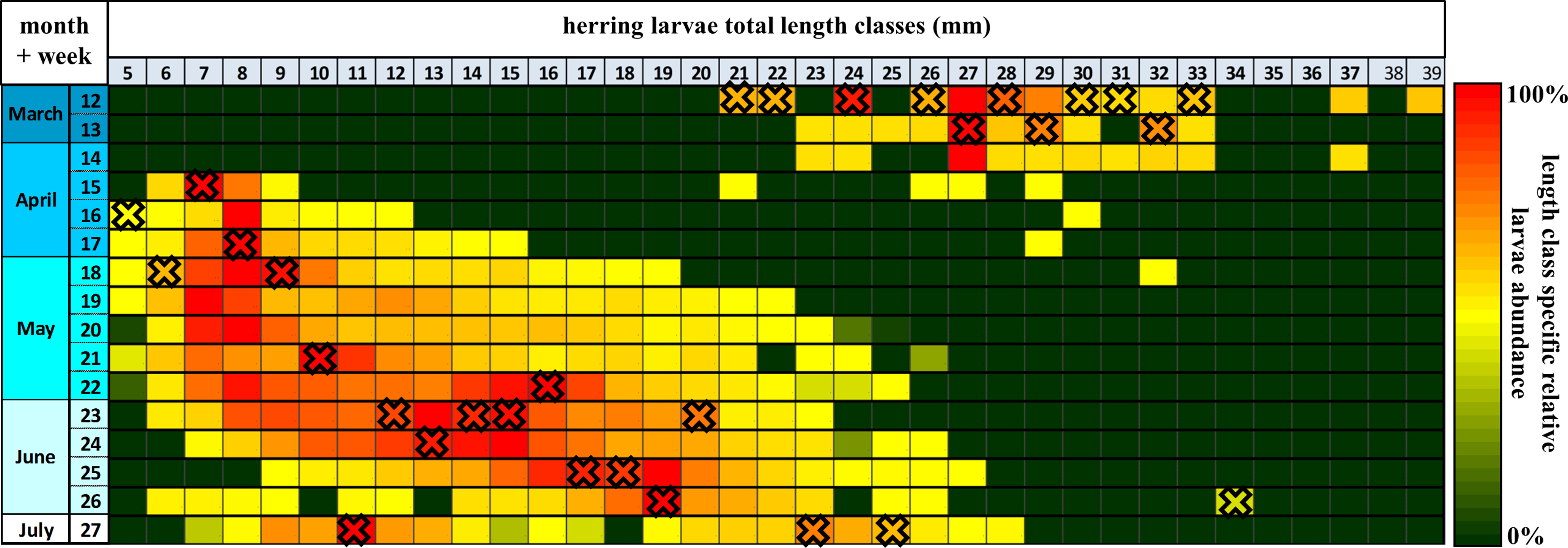


**Figure S4**

**
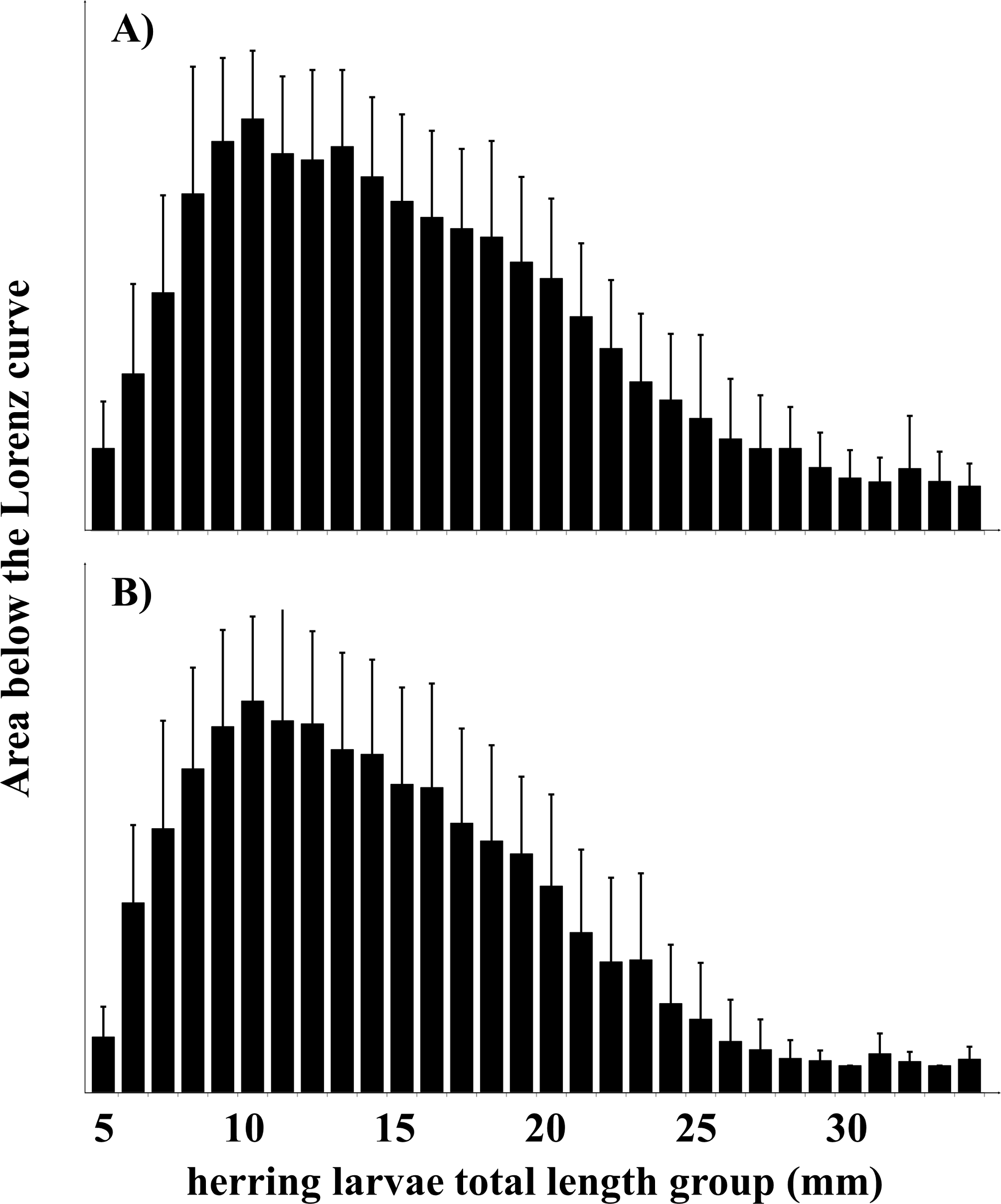
**

**Figure S5**


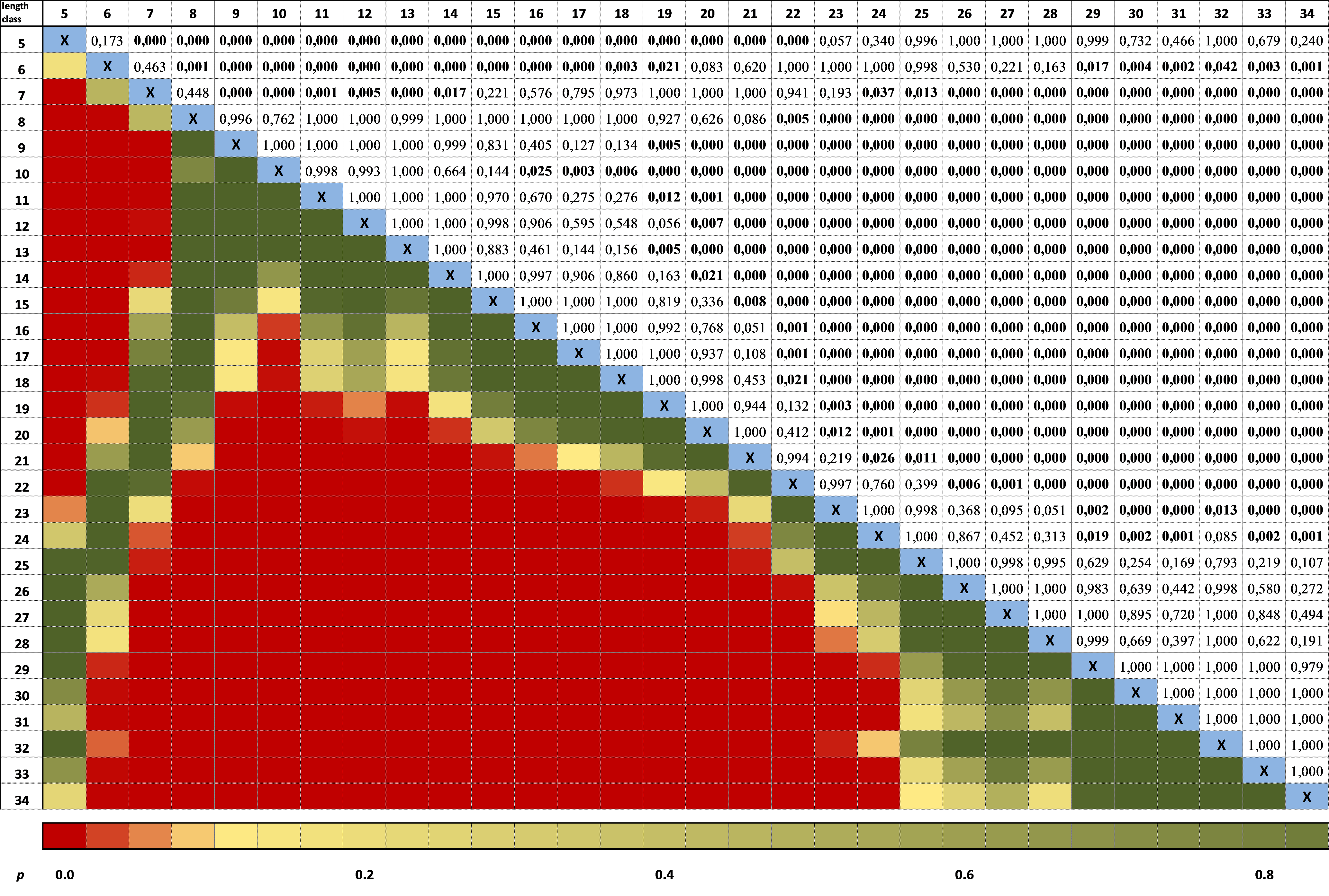


**Figure S6**

**
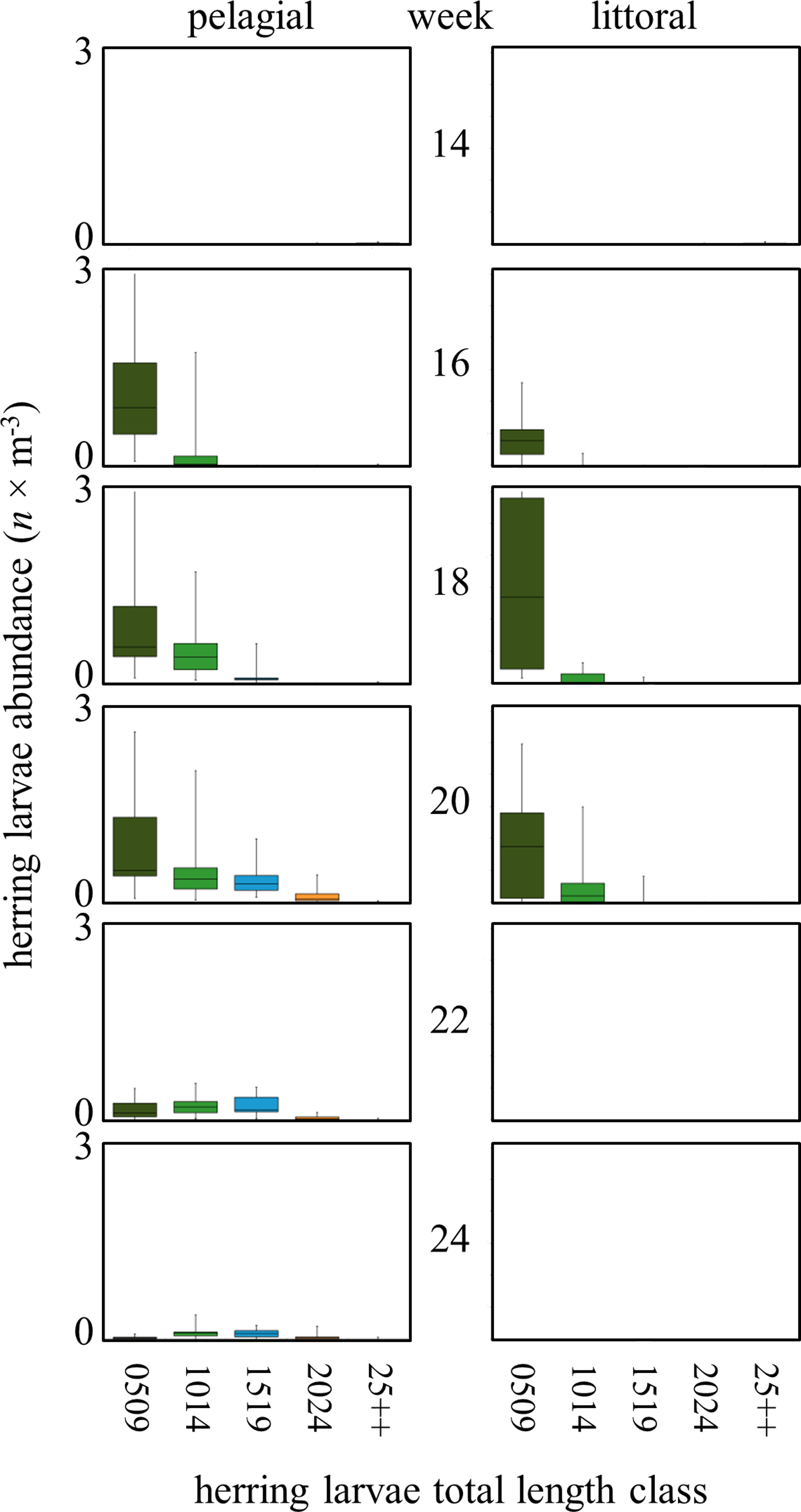
**

**Table S7**
